# *Gynura divaricata* rich in 3, 5−/4, 5-dicaffeoylquinic acid and chlorogenic acid reduces islet cell apoptosis and improves pancreatic function in type 2 diabetic mice

**DOI:** 10.1186/s12986-018-0310-y

**Published:** 2018-10-10

**Authors:** Xiao-Lu Yin, Bing-Qing Xu, Yu-Qing Zhang

**Affiliations:** Silk Biotechnology Laboratory, School of Biology and Basic Medical Sciences, Soochow University, RM702-2303, No. 199, Renai Road, Dushuhu Higher Edu. Town, Suzhou, 215123 People’s Republic of China

**Keywords:** Diabetes mellitus, *Gynura divaricata*, Type 2 diabetes, Fasting blood glucose, Serum insulin, Pancreatic β-cell

## Abstract

**Background:**

Diabetes mellitus is one of the most common chronic diseases that accompanied by severe complications. *Gynura divaricata* (GD), a medicinal and edible plant that is usually used for the treatment of diabetes. Therefore, this study investigates the chemical components of GD with hypoglycemic effect and the possible mechanism lowering blood sugar in T2D diabetic mice.

**Methods:**

The methanol extract of GD was analysed by HPLC-DAD. And then mice with type 2 diabetes induced by a high-fat diet in combination with streptozotocin feed the diet containing lyophilized GD powder for 4 weeks. During this period, fasting blood glucose (FBG) levels and body weight were measured.

**Results:**

GD was rich in four bioactive components of dicaffeoylquinic acid and chlorogenic acid. These components occupied about 2.37% in the GD powder in which the highest level was 3, 5-dicaffeoylquinic acid. Oral GD significantly reduced FBG, fasting serum insulin, and glycosylated serum protein levels, and enhanced antioxidative activities. HE-staining showed that the pathological damage in pancreatic β-cells was ameliorated. An immunohistochemical assay also showed that GD promoted marked pancreatic β-cell regeneration. GD also caused notable increase in GLUT2, GK, MafA, PDX-1, and Bcl-2 as well as reduction in Bax and caspase-3 expression as shown by western blot analysis.

**Conclusions:**

GD exerts the pronounced hypoglycaemic effect by inhibiting islet cell apoptosis and improving pancreatic function. Therefore, GD might have a potential to improve diabetes.

## Background

Diabetes mellitus is one of the most common chronic diseases characterised by metabolic dysfunction and is often accompanied by severe complications [1–3]. Type 2 diabetes mellitus (T2DM) is the most common form of this disease, affecting more than 300 million individuals worldwide [4]. T2DM is a non-insulin-dependent diabetes that is characterized by abnormal insulin secretion and insulin resistance because of pancreatic dysfunction [5]. At present, available therapies have achieved some success include insulin and hypoglycaemic medicines [6]. However, drug resistance and side effects are two aspects to concern. Consequently, researchers are seeking natural products such as traditional Chinese medicinal herbs (TCMH) to prevent or treat diabetes due to their prominent pharmacological activity, low toxicity, and few/no side effects.

*Gynura divaricata* (L.) DC (GD), a kind of TCMH, called “Bai Bei San Qi” in China [7, 8], is a new source of food approved by the Ministry of Health of the People’s Republic of China in 2010 [9]. It is a medicinal and edible plant that is usually used for the treatment of diabetes [8, 10]. The plant contains many biological avtivities, including flavonoids, polysaccharides, phenolic compounds, terpenoids, fatty acids, alkaloids, and cerebrosides [11–15]. It has been used for the treatment of diabetes for a long time in Chinese folk medicine. It was reported that the ethanol extract of GD aerial parts has hypoglycaemic activity in vivo [16, 17]. Some investigations have also demonstrated that both the extract and polysaccharide of GD possess anti-hyperglycaemic activity in diabetic mice and rats [18–20]. Moreover, the fresh leaves of GD can make tea for reducing blood glucose, and could significantly decrease blood glucose in diabetic patients in many areas of China [21]. Importantly, our latest studies also demonstrated the good hypoglycaemic activity of lyophilised powder of GD [22], which successfully demonstrated that GD has the potential to be an efficiently hypoglycaemic medicine. However, the main active components of GD and its function in islet cell apoptosis is rarely reported. Therefore, this investigation was designed to explore the relative mechanisms involved in the modulation of pancreatic function and GD-mediated anti-apoptotic effect.

## Methods

### Materials

The GD was provided by Silk Biotechnology Lab., Soochow University. Fresh GD aerial parts were lyophilised into powders for further research. Other required materials are outlined in the following sections.

### HPLC-MS/MS analysis

High-performance liquid chromatography with diode array detection (HPLC–DAD) was used to analyse sample active components. A total of 250 mg GD lyophilised powder was suspended in 10 mL 70% methanol aqueous solution, which was treated at 75 °C by ultrasonic assisted extraction for 1.5 h. Then, the supernatant was filtered through a 0.22-μm nylon membrane and injected onto a J&K CHEMICA HPLC-C18 reversed-phase column (2.1 69 × 150 mm, 5 μm). The column temperature was maintained at 40 °C, and solvents for the mobile phase were acetonitrile (A) and 0.4% glacial acetic acid (B). The gradient elution was 0 to 30 min; the concentration of B linear gradient was 95 to 70%; 30–50 min, the concentration of B linear gradient maintained 70%. The flow rate was 1.0 mL/min, and the injection volume was 20 μL. The pattern of the eluent was monitored at 323 nm. Mass spectrometric analysis was carried out using a TSQ quantum ultra-triple-quadrupole mass spectrometer (Thermo Fisher Scientific Inc., Waltham, MA, USA), which was equipped with an electro-spray ionisation (ESI) interface in negative mode. The following are the parameters of the mass spectrometer: sheath gas flow rate at 40 (arbitrary units); auxiliary gas flow rate at 10 (arbitrary units); spray voltage at 2500 V; both vaporiser and capillary temperature at 350 °C. Helium was used as the collision gas for collision-induced dissociation (CID).

### Quantification of main bioactive components of GD methanol extract

Quantification of the main bioactive components of GD methanol extract was performed using the external standard method. The HPLC conditions of standards were the same as for the sample. Four standards purchased from Pufei Biotech Co., Ltd. (Shanghai, China) were separately dissolved in 70% methanol aqueous solution. Each standard was prepared into five different concentration gradients, and each standard concentration was analysed by HPLC three times. Then, the four kinds of standard solutions were injected into the HPLC system at various concentration levels. The different concentrations of each standard solution and the corresponding peak areas were recorded. Thus, four standard curves were obtained according to the concentration gradients (*X*) and their corresponding peak areas (*Y*). Ultimately, according to peak areas of sample solution, we can determine the content of each component by standard curves.

### Animals

Healthy male ICR mice (3 weeks of age) were provided by the Experimental Animal Centre in Soochow University, Suzhou, Jiangsu Province. All mice had free access to water and diet and were housed under standard conditions (18 °C–22 °C, humidity 50–80%, with a cycle of 12–12 h light/dark). After 3 days of acclimation, the mice were divided into two groups: a normal group and a type 2 diabetic group (untreated diabetic group). Mice in the normal group were continually fed normal food (chow diet). Type 2 diabetic mice were fed with a diet containing 20% sucrose, 18% lard, 3% egg yolk, and 59% basal feed (high-fat diet, HFD). After 4 weeks of HFD feeding, these mice were fasted for 12 h (with free access to water), and each mouse was injected once with low-dose streptozotocin (STZ; Sigma, USA) at 100 mg/kg. Seven days after the injection, blood samples of mice were withdrawn from the tail vein, and fasting blood glucose (FBG) was determined; mice with more than 11.1 mmol/L of FBG were considered to be diabetic and included in the study.

### Experimental design

The HFD- and STZ-treated mice that developed diabetes were randomly divided into four groups with each 10 mice: the diabetic model group (HFD + STZ group, shorten as model group) and three GD-treated diabetic groups with doses of 1, 5 and 10%. Finally, the normal mice and the diabetic model mice received a normal diet (chow diet). The three groups of GD-treated diabetic mice feed the normal diets containing 1, 5 and 10% GD, respectively. The administration was given daily for 4 weeks. After 4-week treatment, the eyeballs of mice were extracted, and blood was drawn and then sacrificed. Their pancreas was excised. All animal experiments abided by the rules of the international animal welfare committee requirements and regulations. All animal experimental protocols used in this study were approved by the Animal Ethics Committee at Soochow University (201504A136).

### FBG detection

During the 4 weeks of GD treatment, FBG was determined using a Blood Glucose Meter (ONETOUCH, LifeScan, Inc., Milpitas, CA, USA) once a week. The hypoglycaemic rates of these groups were calculated by the following formula: (FBG before administration–FBG after administration)/FBG before administration× 100%.

### Oral glucose tolerance test and insulin tolerance test

For oral glucose tolerance test (OGTT), ICR mice were fasted overnight for 12 h. And a small tail cut was made to measure the blood glucose by OneTouch glucometer (from Johnson & Johnson Medical (Shanghai) Ltd., China) as starting blood glucose value(0 min), Following an oral feeding of glucose (2 g/kg mice body weight), blood glucose was continuously measured at 30 min, 60 min, 90 min and 120 min. For the insulin tolerance test (ITT) with mice would be fasting 4 h and injection of insulin (1 IU/kg mice body weight). Blood glucose levels were determined by tail vein sampling at the indicated intervals (0, 15, 45, 60, 90 min) using OneTouch glucometer.

### Fasting serum insulin detection

The blood was left at room temperature for about 2 h and then the serum was obtained by centrifugation. The assay was performed using a mouse ELISA kit (Nanjing Jiancheng Bioengineering Institute, Nanjing, China). All protocols were carried out according to the manufacturer’s instructions.

### HOMA-IR and ISI

Homeostasis model assessment of insulin resistance (HOMA-IR) was calculated using the following formula [23, 24]: [FBG (mmol/L) × fasting plasma insulin (mIU/L)]/22.5. HOMA-IR has been widely accepted to evaluate the insulin resistance in patients with diabetes. Insulin sensitivity index (ISI) was computed according to the following formula: Ln[1/(FBG × fasting plasma insulin)] [25].

### GSP concentration detection

The blood was taken from the mouse’s eyeballs and left at room temperature for about 2 h and then the serum was obtained by centrifugation. Glycosylated serum protein (GSP) was determined using a commercial kit purchased from Nanjing Jiancheng Bioengineering Institute (Nanjing, China) according to the manufacturer’s instructions.

### Measurement of levels of T-SOD, GSH-PX and MDA in pancreatic tissue

80 mg of pancreatic tissues were accurately weighed, then it was added 900 μL ice normal saline and stored − 80 °C. The cold tissue were homogenized to obtain 10% of the pancreatic tissue of slurry. After centrifugation, the supernatant was used to evaluate T-SOD and GSH-PX activities and MDA level in pancreas homogenates of mice. All the test kits were from the Nanjing Jiancheng Bioengineering Institute, Nanjing, China.

### Histopathologic examination

The pancreas was quickly removed from the mice, washed with normal saline, and then dried, weighed, cut into small pieces, and finally fixed with 10% formalin. The tissue dehydration was performed with increasing concentrations of acetone. The pancreas samples were sectioned and stained with haematoxylin and 3-μm thickness of slices were then cut on an HM340E microtome, stained with H&E and imaged histocyte structure under an optical microscope (U-III Multi-point Sensor System; Nikon, Tokyo, Japan).

### Immunohistochemical examination

To detect insulin protein, sections were incubated overnight with anti-insulin antibody from Boster Bio-engineering Limited Company (Wuhan, China) and then visualisation using the complex method of avidin-biotin peroxidase. An optical microscope was used to acquire images.

### Western blot analysis

To detect the expression levels of glucose transport protein 2 (GLUT2, 1:1000), glucokinase (GK, 1:1000), v-maf musculoaponeurotic fibrosarcoma oncogene family protein A (MafA, 1:1000), pancreatic duodenal homeobox-1 (PDX-1, 1:1000), B-cell lymphoma-2 (Bcl-2, 1:1000), Bcl2-associated X (Bax, 1:1000), and cysteinyl aspartate specific proteinase-3 (caspase-3, 1:1000), proteins isolated from the pancreatic tissues were separated by 10% sodium dodecylsulfate–polyacrylamide gels and then transferred to a polyvinylidene fluoride membrane (Millipore, Shanghai, China). The membrane was incubated overnight with desired primary antibodies at 4 °C. After washing, the membrane was incubated for 1 h with HRP-conjugated secondary antibodies (1:5000). Protein was visualised with an enhanced chemiluminescence detection kit. Protein expression levels were normalised using GAPDH (1:3000) as the internal standard. Bands were visualized using a UVP detection system. Band intensity was quantified using LAB WORKS4.6.

### Statistics

The experimental data are presented as mean ± standard deviation (SD). Significant differences between two sets of data were assessed using one-way ANOVA (Origin 7.5 version). A value of *P* < 0.05 was considered statistically significant.

## Results

### Identification of chemical components of GD

**Fig. 1:**
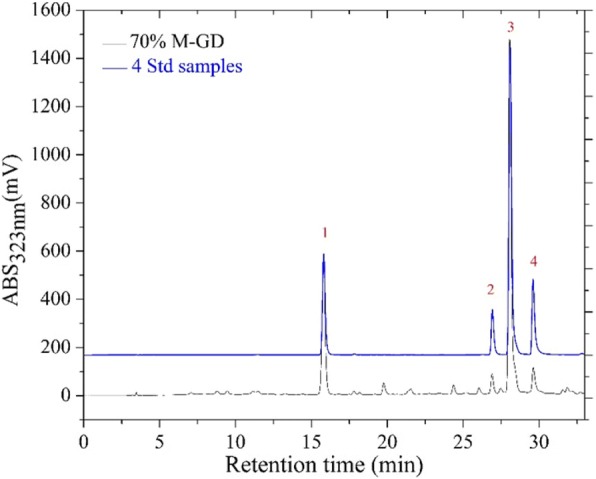
HPLC chromatogram of 70% methanol extract of GD and four standards at 323 nm. 70% M-GD, 70% methanol extract of GD lyophilised powder; 4Std, 4 standards including chlorogenic acid (1), 3,4-dicaffeoylquinic acid (2), 3,5-dicaffeoylquinic acid (3) and 4,5-dicaffeoylquinic acid (4)

**Fig. 2:**
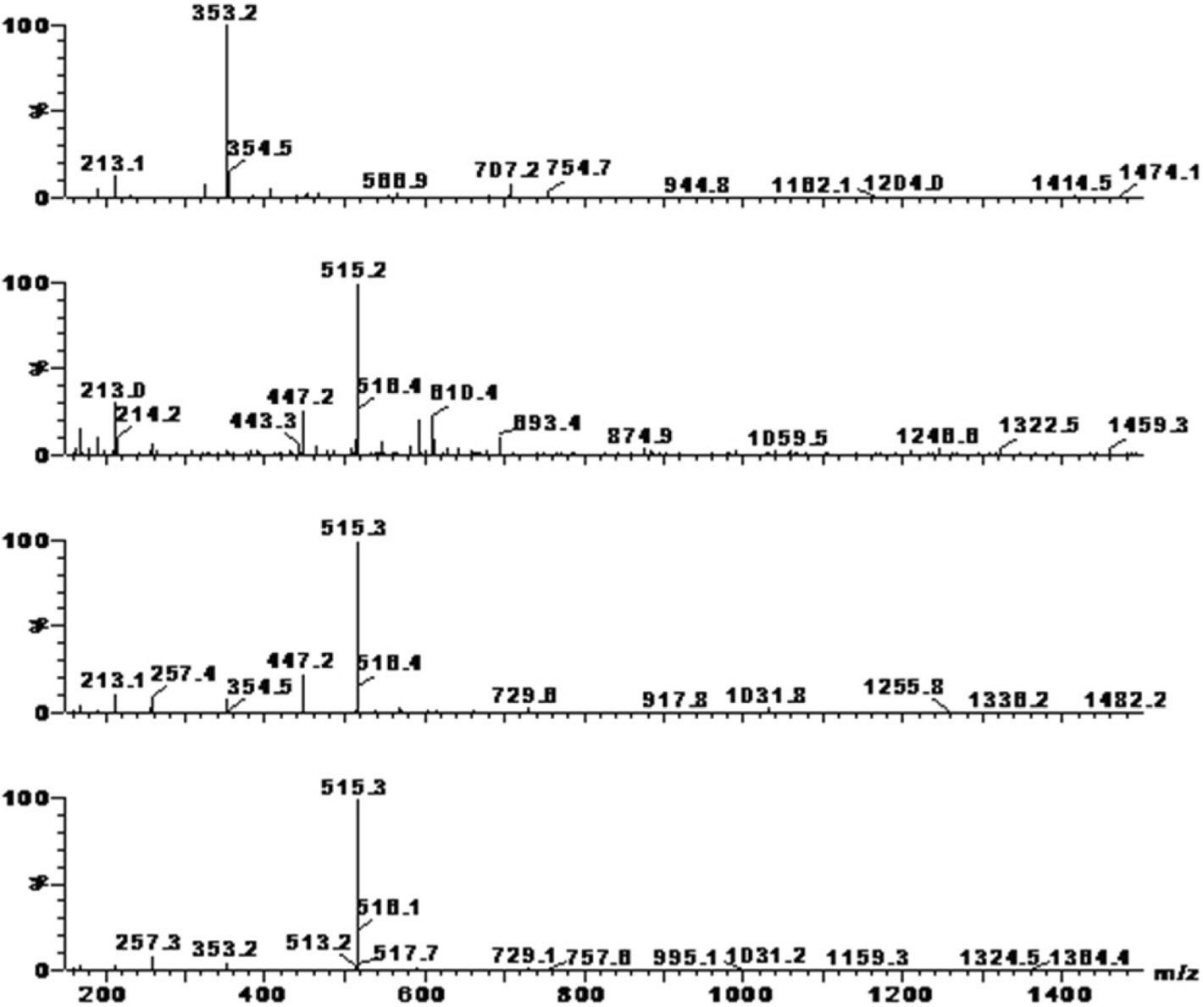
Negative ion mass spectra of four main chemical constituents in 70% methanol extract of GD

**Fig. 3:**
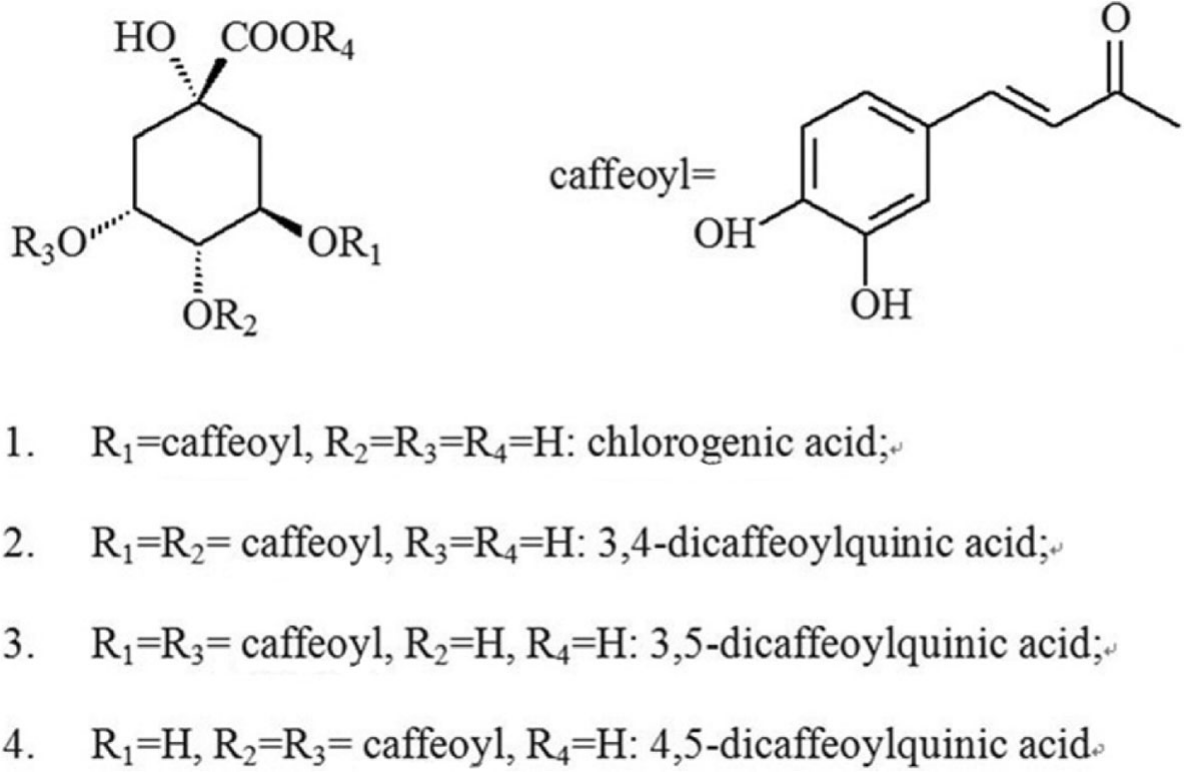
Structures of chlorogenic acid, 3,4-dicaffeoylquinic acid, 3,5-dicaffeoylquinic acid and 4,5-dicaffeoylquinic acid

Four components were isolated and identified by HPLC-MS/MS. According to the HPLC chromatogram (Fig. 1) and the mass spectrum data (Fig. 2), the four peaks were identified as chlorogenic acid (3-caffeoylquinic acid); 3, 4-dicaffeoylquinic acid; 3, 5-dicaffeoylquinic acid; and 4, 5-dicaffeoylquinic acid (Table 1). Figure 1 indicates that high amounts of chlorogenic acid, 3,4-dicaffeoylquinic acid, 3,5-dicaffeoylquinic acid, and 4,5-dicaffeoylquinic acid are present in a 70% methanol extract of GD. Structures of these four compounds are shown in Fig. 3.

**Table 1:** HPLC/MS analysis of 70% methanol extract of GD

Peak	Retention time (min)	[M-H]^−^	Molecular Weight	Compound name
1	15.790	353.2	354	chlorogenic acid
2	26.893	515.2	516	3, 4-dicaffeoylquinic acid
3	28.060	515.3	516	3, 5-dicaffeoylquinic acid
4	29.615	515.3	516	4, 5-dicaffeoylquinic acid

### Quantification of main bioactive components of GD methanol extract

A quantitative determination of the four main bioactive components of GD methanol extract was performed by HPLC-DAD. The results are shown in Table 2. The contents of chlorogenic acid, 3,4-dicaffeoylquinic acid, 3,5-dicaffeoylquinic acid, and 4,5-dicaffeoylquinic acid in GD powder calculated according to the linear regression equation of each standard are 5.57 ± 0.35 mg/g, 0.67 ± 0.08 mg/g, 10.29 ± 0.74 mg/g, and 6.84 ± 0.14 mg/g, respectively.

**Table 2:** Quantification of four bioactive components in methanol extract of GD

Bioactive components	Linear regression equations	Correlation coefficients (R^2^)	Contents (mg/g GD powder)
Chlorogenic acid	*y* = 381,854.86597 + 57,440,112.13043*×*	0.99884	5.57 ± 0.35
3, 4-dicaffeoylquinic acid	*y* = −27,745.44883 + 68,618,107.60542*×*	0.99988	0.67 ± 0.08
3, 5-dicaffeoylquinic acid	*y* = 250,500.51458 + 78,350,260.14366*×*	0.99905	10.29 ± 0.74
4, 5-dicaffeoylquinic acid	*y* = 26,676.51400 + 9,837,283.14720*×*	0.99975	6.84 ± 0.14

### Effect of GD on FBG

The findings in Table 3 showed that FBG levels in the diabetic model group were much higher than those in normal (control) mice (*P* < 0.01). Notably, the three GD-treated diabetic groups effectively led to an obvious reduction of FBG in a dose-depended manner by 4 weeks of GD treatment (*P* < 0.05, P < 0.01). With the addition of GDP, the blood glucose of mice gradually decreased; the FBG of 10% GD group approached to 12.44 mmol/L. Moreover, the hypoglycaemic rate of 1% GD group was 21.07% and the hypoglycaemic rate of the highest dose group has peaked at 41.4%. The ultimate hypoglycaemic rates of the three GD-treated diabetic groups also directly proved that GD could efficiently lower FBG level.

**Table 3:** Effect of GD on FBG

Groups	FBG (mmol/L)					Hypoglycae-mic rate
	0 Week	1 Week	2 Week	3 Week	4 week	
Normal	5.36 ± 1.22	6.80 ± 0.35	5.34 ± 0.96	5.76 ± 0.81	5.47 ± 0.89	–
Model	21.94 ± 4.63^**^	20.88 ± 3.53^**^	21.20 ± 4.55^**^	19.85 ± 2.76^**^	22.78 ± 0.87^**^	–
1% GD	22.07 ± 0.70	19.52 ± 5.33	16.38 ± 4.54^#^	17.40 ± 3.85	17.42 ± 5.76^#^	21.07%
5% GD	22.06 ± 0.55	18.39 ± 2.98	13.97 ± 3.75^##^	14.99 ± 4.69^#^	13.39 ± 5.78^##^	39.30%
10% GD	21.23 ± 1.58	17.49 ± 4.92	12.47 ± 5.32^##^	13.24 ± 5.38^##^	12.44 ± 4.60^##^	41.40%

1% GD, 1% GD-treated diabetic group; 5% GD, 5% GD-treated diabetic group; 10% GD, 10% GD-treated diabetic group. The experimental data are presented as means ± SD. ^**^P < 0.01, versus normal group; ^#^*P* < 0.05 and ^##^ *p* < 0.01, respectively, versus diabetic model group. The hypoglycaemic rate = (FBG before treatment-FBG after 4 weeks of GD treatment)/FBG before treatment×100%

### Effect of GD on OGTT and ITT

**Fig. 4:**
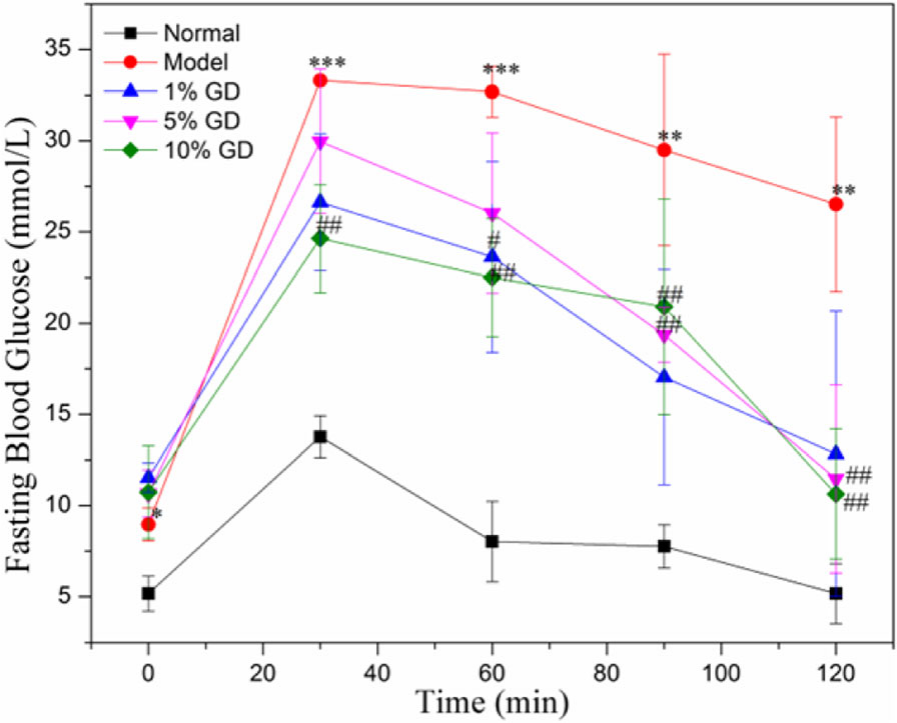
The effect of different dose of GDP on OGTT in mice

**Fig. 5:**
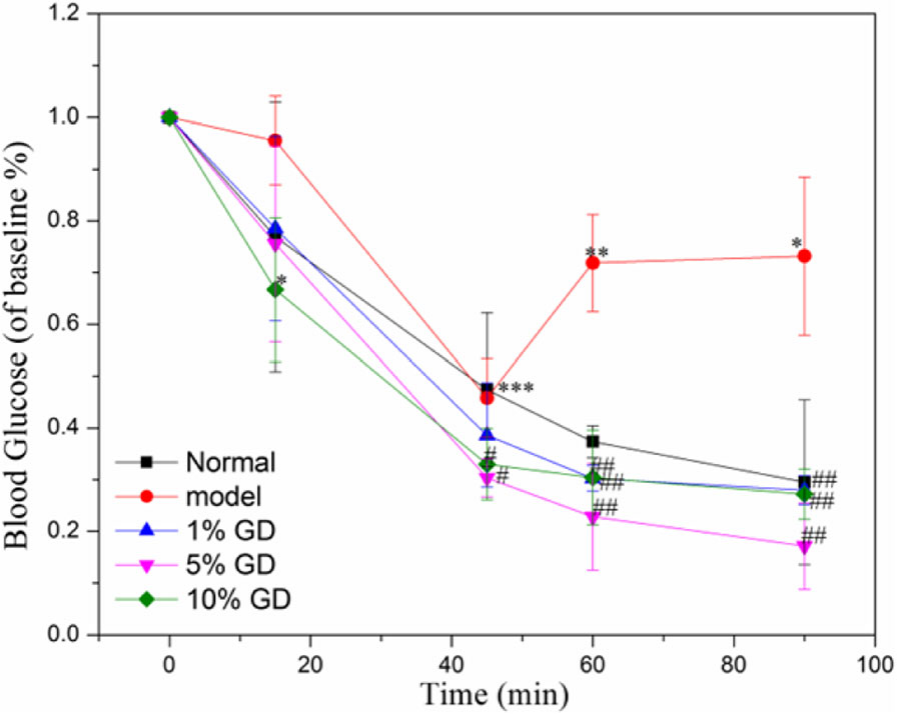
The effect of different dose of GDP on ITT in mice

In order to OGTT, the blood glucose level after oral glucose was determined at 30 mim, 60 min, 90 min and 120 min. In normal group, the final blood glucose was the same as the initial blood glucose. And the blood glucose still very high in model group mice (Fig. 4). Interestingly, the blood glucose had evidently decrease in high dose GD in comparison with the lower dose GD and in a dose-dependent manner, suggesting that glucose-lowering effect of GD treatment was rapid and stable. The OGTT results showed that GD treatment could improve glucose tolerance. Insulin resistance was demonstrated in ITT. In the ITT, the GD-treated groups reduced the blood glucose level at 15 min, 45 min and 60 min (Fig. 5), as demonstrated by lower blood glucose 15 min to 60 min following insulin injection, compared to the model mice. The result suggested that high-dose GD improved insulin sensitivity and insulin resistance.

### Effect of GD on fasting serum insulin

**Fig. 6:**
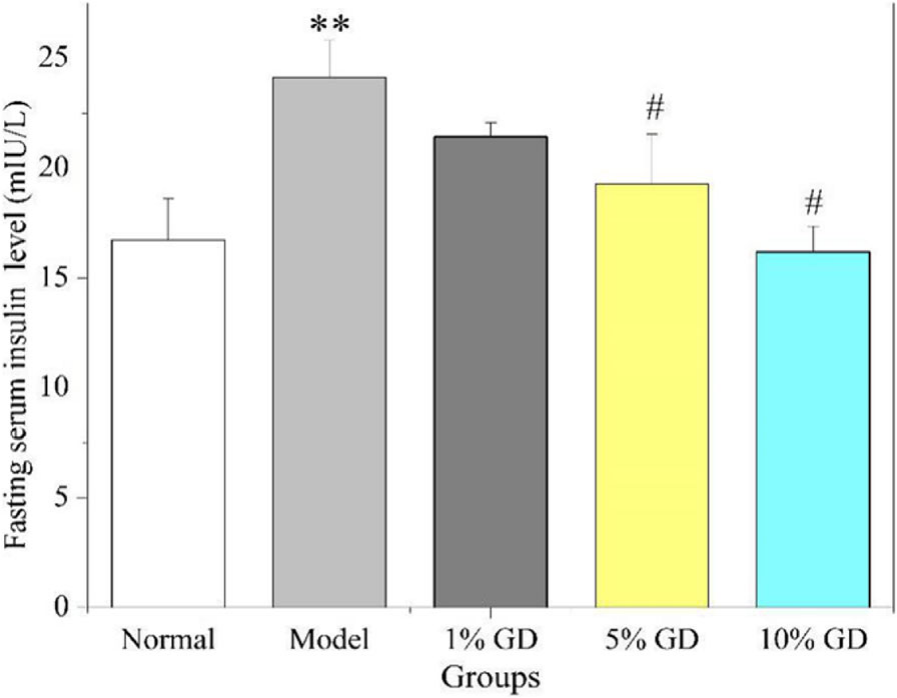
Effect of oral GD on the fasting serum insulin levels. The fasting serum insulin levels were determined in mice in the fasting state after 4 weeks of GD treatment. 1% GD, 1% GD-treated diabetic group; 5% GD, 5% GD-treated diabetic group; 10% GD, 10% GD-treated diabetic group. The experimental data are presented as means ± SD. ^**^P < 0.01, versus normal group; ^#^*P* < 0.05, versus diabetic model group

T2DM was characterised by insulin resistance and a higher serum insulin concentration in the HFD- and STZ-induced diabetic mice [26]. Furthermore, insulin resistance is often accompanied by compensatory hyperinsulinemia and hyperglycaemia [27]. We attempted to assess the changes in fasting serum insulin. The findings, shown in Fig. 6, indicated that the fasting serum insulin level of the diabetic model mice was markedly increased compared with those of the normal mice (*P* < 0.01). Following the GD treatment, the fasting serum insulin levels in diabetic mice of three GD-treated groups decreased evidently with a dose-dependent manner. The statistic data showed that 5 and 10% GD groups had significant differences compared with the diabetic model group (*P* < 0.05). The result demonstrated that GD could efficiently restrain an abnormal increase of insulin secretion and ameliorate the insulin resistance.

### Effects of GD on ISI and HOMA-IR

**Fig. 7:**
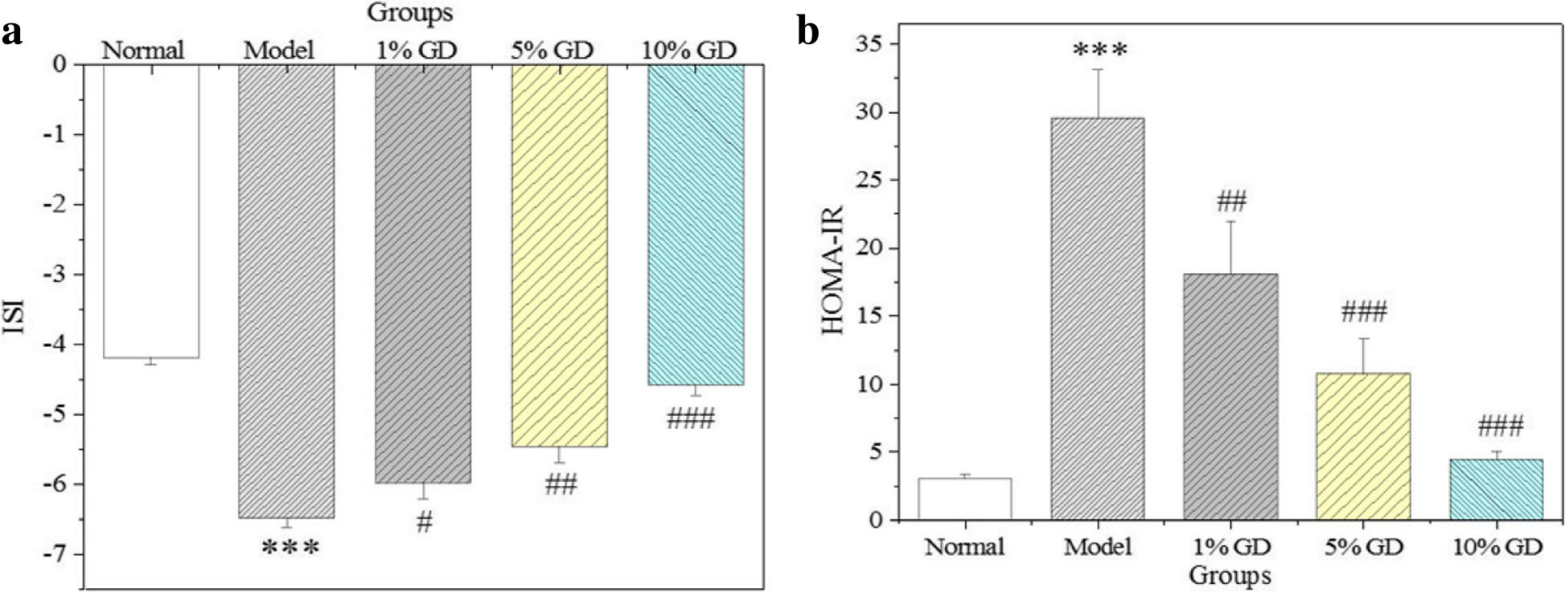
Effects of GD on ISI (**a**) and HOMA-IR (**b**). 1% GD, 1% GD-treated diabetic group; 5% GD, 5% GD-treated diabetic group; 10% GD, 10% GD-treated diabetic group. The experimental data are presented as means ± SD. ^***^P < 0.01, versus normal group; ^#^*P* < 0.05, ^##^*p* < 0.01 and ^###^*p* < 0. 001, respectively, versus diabetic model group

The insulin metabolism of mice was evaluated by an analysis of ISI and HOMA-IR. Insulin resistance was subsequently triggered because of the abnormally increased serum insulin level of the diabetic model group. As shown in Fig. 7, compared to the normal group, we observed an obvious decrease in ISI (*P* < 0.001) and a significant increase in HOMA-IR, (P < 0.001) in the diabetic model group. Importantly, oral administration of GD led to a prominent reduction in the HOMA-IR index, which in turn progressively increased the ISI index. These results suggested that insulin resistance was ameliorated in diabetic mice after the administration of GD, indicating that GD can enhance insulin sensitivity of type 2 diabetic mice.

### Effect of GD on GSP

**Fig. 8:**
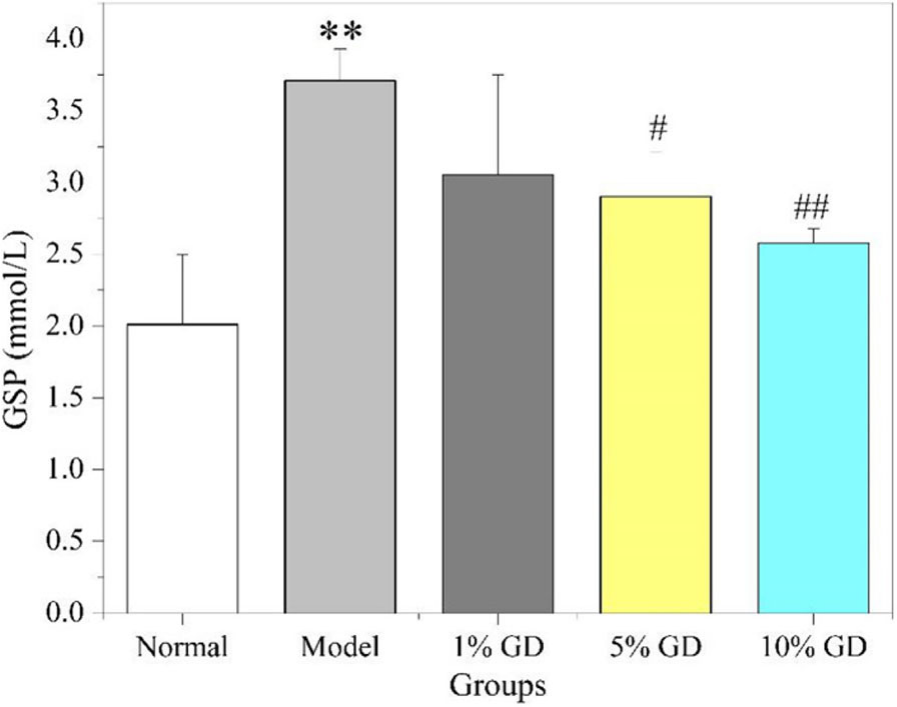
Effect of GD on GSP. 1% GD, 1% GD-treated diabetic group; 5% GD, 5% GD-treated diabetic group; 10% GD, 10% GD-treated diabetic group. The experimental data are presented as means ± SD. ^**^P < 0.01, versus normal group; ^#^*P* < 0.05 and ^##^*p* < 0.01, respectively, versus diabetic model group

GSP, which is related to the blood glucose concentration, can effectively reflect the average blood glucose level of diabetic patients in the past 1 to 2 weeks. Figure 8 shows that the GSP level (3.7 mmol/L) in diabetic model mice was obviously increased relative to that of normal mice (*P* < 0.01). Strikingly, GD administration significantly reversed GSP levels in 5% GD-treated diabetic group (*P* < 0.05) and 10% GD-treated diabetic group (P < 0.01). The 10% GD-treated group had reduced to 2.5 mmol/L, and indirectly confirmed that GD has a good effects on lowering blood glucose.

### Effects of GD on GSH-PX, T-SOD activities, and MDA content

Table 4 illustrates GSH-PX, T-SOD activities, and MDA content in the pancreas of experimental mice. As expected, the activities of GSH-PX and T-SOD in pancreatic tissue of diabetic model group were dramatically reduced and MDA content significantly increased when compared to normal group (P < 0.01). The level of GSH-PX and T-SOD in the high dose group has reached 76.05 U/mgprot and 197.18 U/mgprot, respectively. Remarkably, oral administration of GD prompts the enzyme activities and decreases MDA content in a dose-dependent manner. These results suggest that GD may enhance antioxidant capacity effectively and reduce oxidative damage.

**Table 4:** Effects of GD on GSH-PX, T-SOD and MDA

Groups	GSH-PX (U/mgprot)	T-SOD (U/mgprot)	MDA (nmol/mgprot)
Normal	81.57 ± 5.48	210.50 ± 2.37	0.68 ± 0.10
Model	49.68 ± 6.89^**^	131.99 ± 10.19^**^	1.57 ± 0.31^**^
1% GD	61.10 ± 6.34	138.79 ± 3.78^#^	1.24 ± 0.19
5% GD	68.43 ± 7.49^#^	184.35 ± 10.02^##^	0.97 ± 0.09^#^
10% GD	76.05 ± 5.31^##^	197.18 ± 7.74^##^	0.79 ± 0.10^#^

1% GD, 1% GD-treated diabetic group; 5% GD, 5% GD-treated diabetic group; 10% GD, 10% GD-treated diabetic group. The experimental data are presented as means ± SD. ^**^P < 0.01, respectively, versus normal group; ^#^*P* < 0.05 and ^##^*P* < 0.01, respectively, versus diabetic model group

### Histopathologic examination

**Fig. 9:**
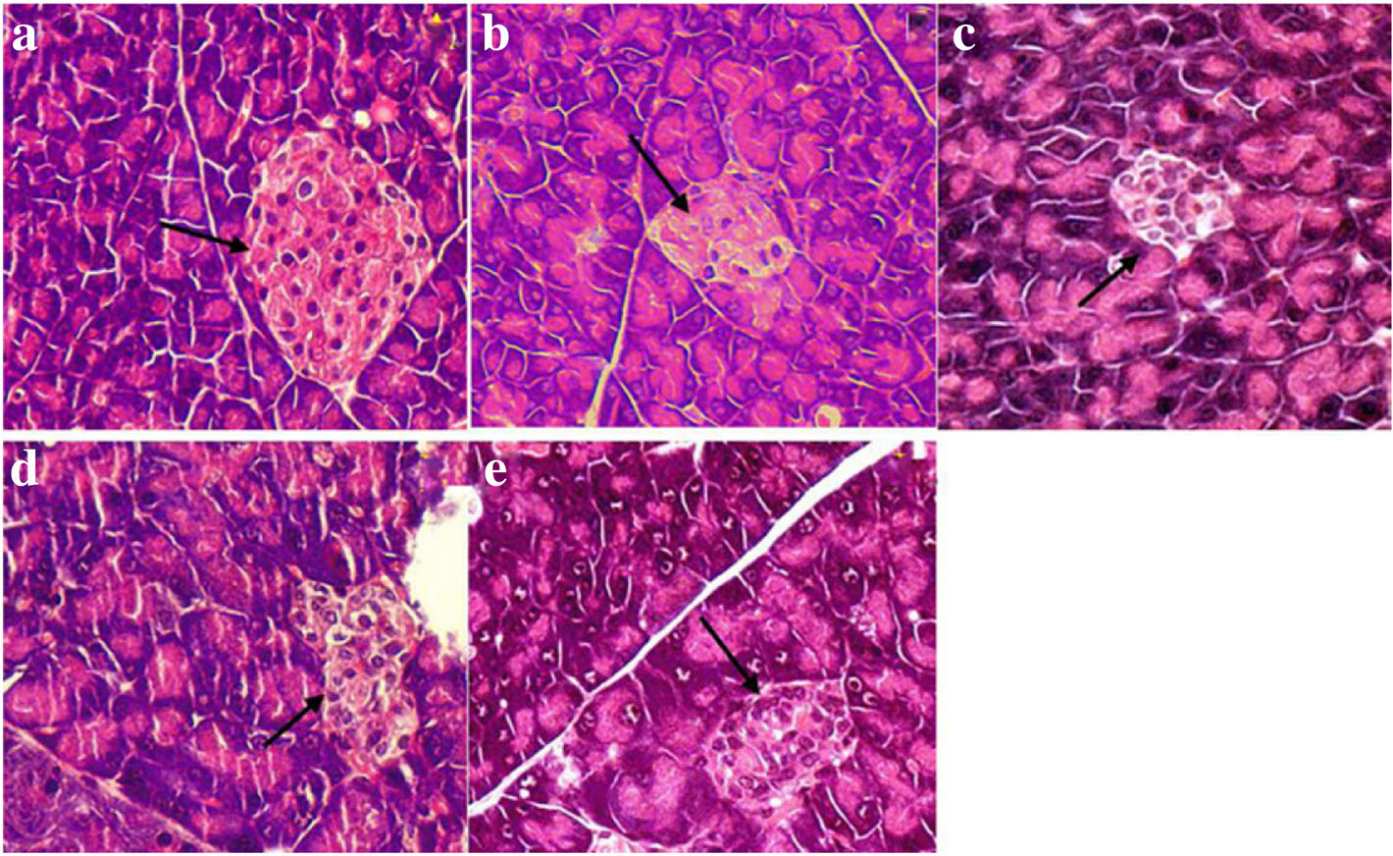
Histopathologic examination of pancreas in diabetic mice (HE stain, × 400). **a** normal group; **b** diabetic model group; **c** 1% GD-treated diabetic group; **d** 5% GD-treated diabetic group; **e** 10% GD-treated diabetic group. Arrows represent the pancreatic islet with pancreatic β-cells

To evaluate the histopathologic alterations in the pancreatic tissue, H&E staining analysis was performed. A complete pancreatic islet structure of normal mice displaying regularly distributed and abundant pancreatic β-cells was observed (Fig. 9a). In contrast, the islets of diabetic model mice were atrophic and severely damaged and were accompanied by an obvious reduction of pancreatic β-cells (Fig. 9b). Nevertheless, the histopathologic changes in the islets exhibited a marked recovery with the increase of pancreatic β-cell counts and the improvement of islet structure in a dose-dependent manner after 4 weeks of GD treatment. The pancreases of mice from the 10% GD group had no obvious pathological changes or hollowing, and the complete islet structure could be observed (Fig. 9e). The result suggested that GD effectively improved the damaged islet structure in diabetic mice.

### Immunohistochemical examination

**Fig. 10:**
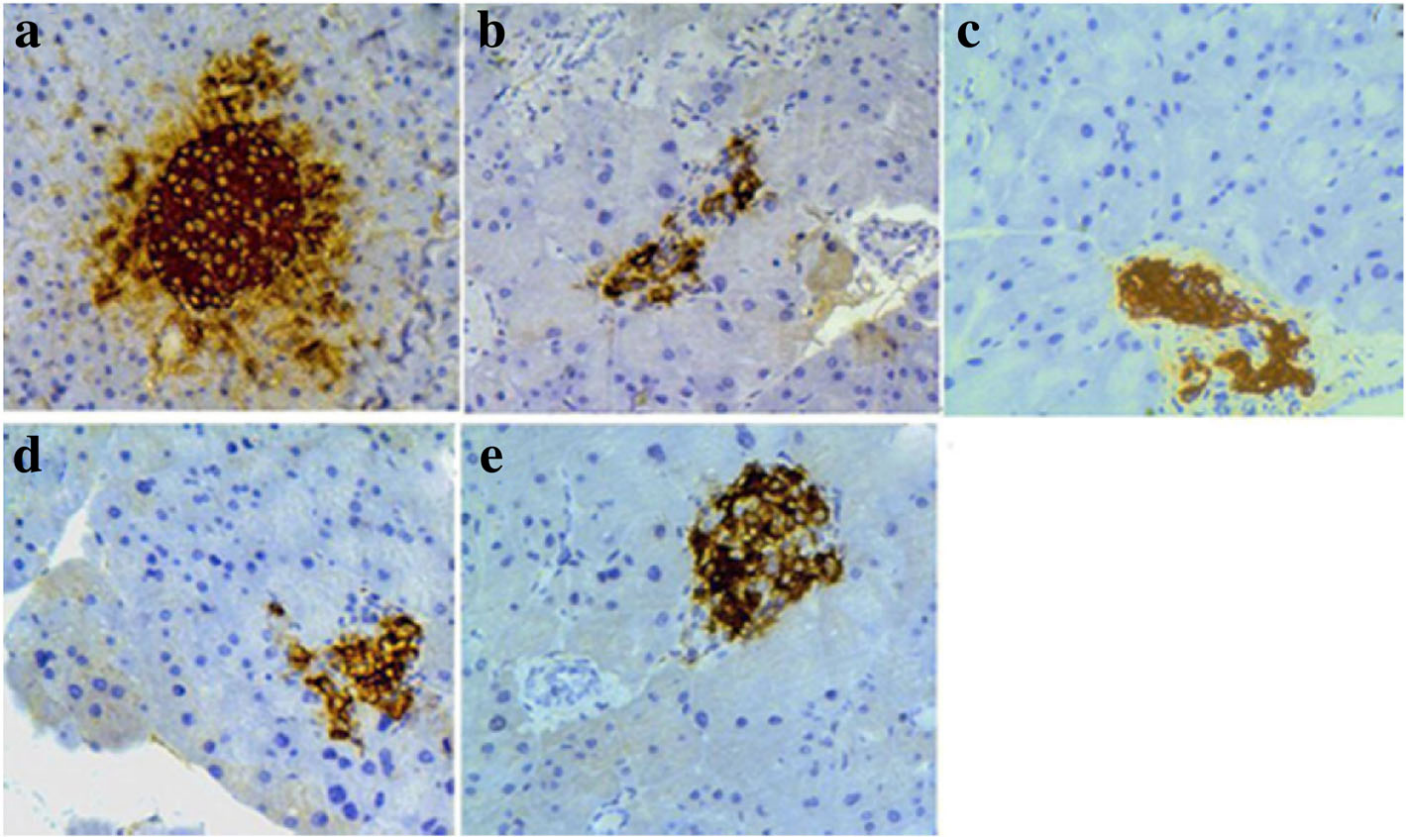
Insulin immunohistochemistry of pancreas in diabetic mice (× 400). **a** normal group; **b** diabetic model group; **c** 1% GD-treated diabetic group; **d** 5% GD-treated diabetic group; **e** 10% GD-treated diabetic group

As shown in Fig. 10, a large number of insulin-positive β-cells were found in pancreatic islets of normal mice (Fig. 10a). On the contrary, the number of insulin-positive β-cells was conspicuously lessened in pancreatic islets of diabetic model mice (Fig. 10b). However, this condition was significantly reversed in GD-treated diabetic groups and showed a dose-depended effect by 4 weeks of GD treatment (Fig. 10c, d, e), The distribution area of the brown granules was larger and formed on oval shape in the 10% GD group. The results demonstrated that GD treatment can effectively improve islet function and potentiate insulin secretion in diabetic mice.

### Effects of GD on some key protein expressions in diabetic mice

**Fig. 11:**
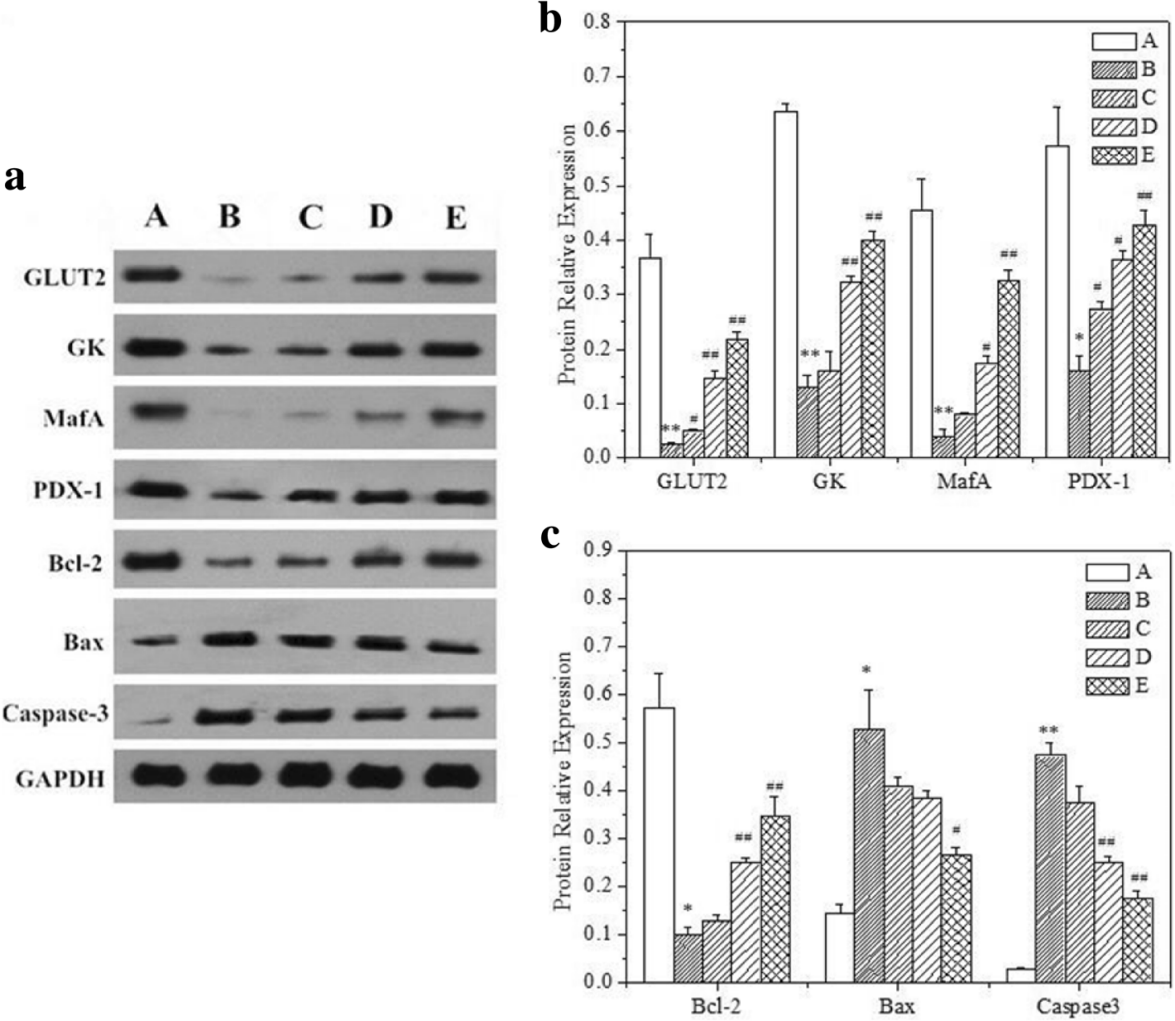
Effect of GD on GLUT2, GK, MafA, PDX-1, Bcl-2, Bax, and caspase-3 protein expressions in pancreatic tissues. (A) Western blot analysis of GLUT2, GK, MafA, PDX-1, Bcl-2, Bax, and caspase-3 protein expressions. (B) Quantitative analysis of GLUT2, GK, MafA, and PDX-1 protein expressions. (C) Quantitative analysis of Bcl-2, Bax, and caspase-3 protein expressions. A: normal group; B: diabetic model group; C: 1% GD-treated diabetic group; D: 5% GD-treated diabetic group; E: 10% GD-treated diabetic group. The experimental data are presented as means ± SD. ^*^P < 0.05 and ^**^P < 0.01, respectively, versus normal group; ^#^P < 0.05 and ^##^P < 0.01, respectively, versus diabetic model group

Finally, we examined the effects of GD on the expression of key proteins involved in the regulation of pancreatic function and cell apoptosis. As shown in Fig. 11, the diabetic model group exhibited an evident decrease in the expression of GLUT2, GK, MafA, PDX-1, and Bcl-2 and an increase in the expression of Bax and caspase-3 in the pancreatic tissue when compared with normal mice. These results were consistent with the results of pancreas pathologic tissue. Further, this effect was gradually reversed in a dose-dependent manner with addition of three different doses of the GD diet and the 10% GD group had the best effects. Overall, these results suggest that a GD diet might contribute to inhibiting the apoptosis of pancreatic cells and preserving pancreatic functionality.

## Discussion

**Fig. 12:**
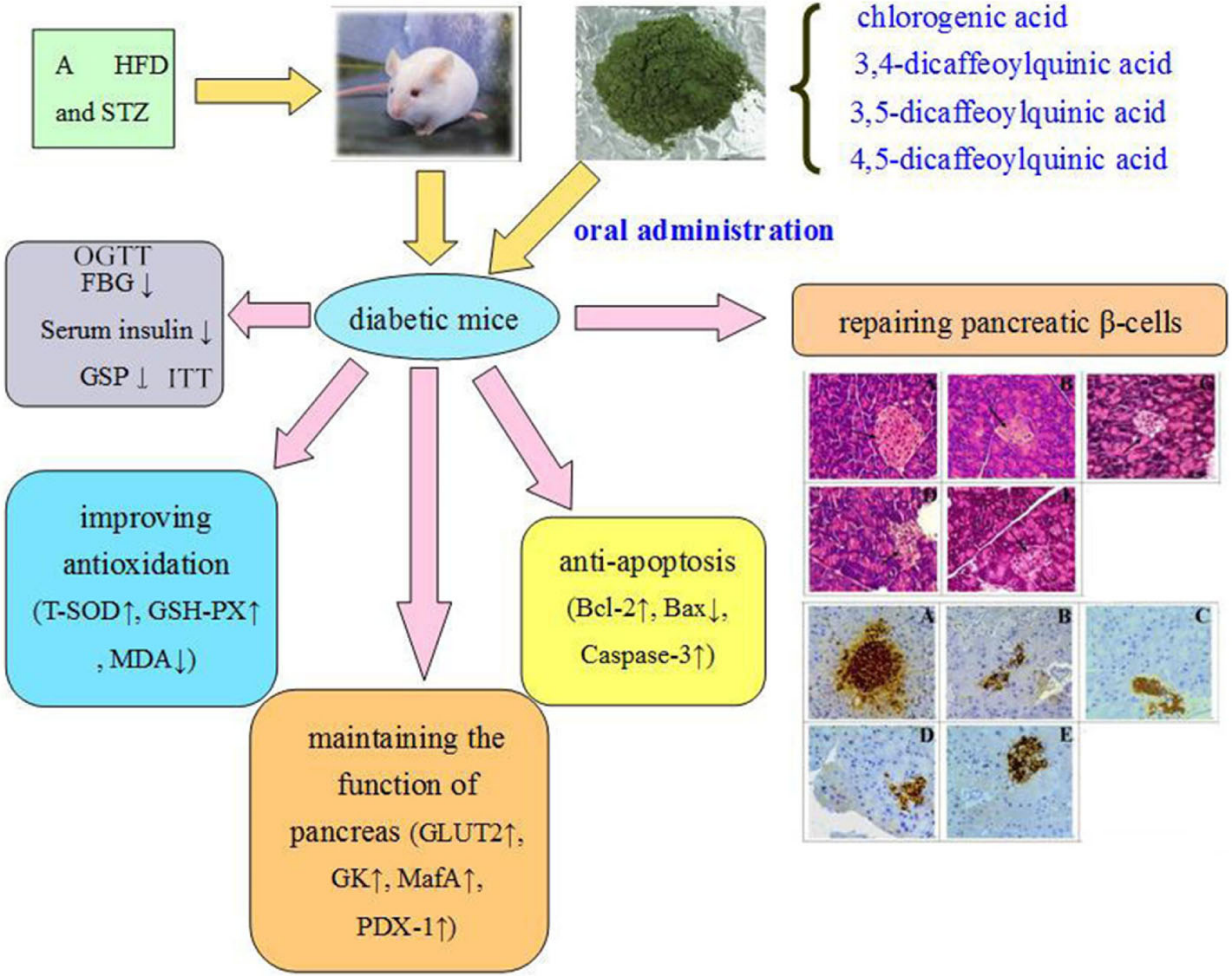
Schematic representation of the protective effect of GD diet in pancreatic tissue of type 2 diabetic mice

GD is an edible medicinal plant, and the roots, stems, and leaves can be used as medicine. The plant was found to have high amounts of crude protein, crude fibre, vitamin C, and minerals, which demonstrates the plant’s nutritional value as a vegetable. In addition, the plant has been used for the treatment of diabetes and other diseases for a long time. In the present study, we examined the protective effect of GD against a HFD and STZ-induced type 2 diabetic mice and its underlying mechanism in pancreatic tissue, which provided a further explanation for the therapeutic effects of GD on T2DM (Fig. 12).

GD extract was analysed by HPLC-DAD chromatography, and abundant chlorogenic acid and its derivatives were found (Fig. 1). The result of the quantification showed that the contents of four bioactive components in the GD were about 2.37%. And the contents follow the sequence of 3, 5-dicaffeoylquinic acid > 4, 5-dicaffeoylquinic acid > chlorogenic acid > 3, 4-dicaffeoylquinic acid. Chlorogenic acid and dicaffeoylquinic acid are esters of one or two caffeic acid(s) with a quinic acid. Caffeic acid and quinic acid are long known antioxidants [28]. Caffeoylquinic acids are also characterised as natural antioxidants [9], and 3,4-dicaffeoylquinic acid and 3,5-dicaffeoylquinic acid isolated from GD have a significant inhibitory effect on protein tyrosine phosphatase 1B [9]. 3,4-dicaffeoylquinic acid and 4,5-dicaffeoylquinic acid isolated from this plant showed obvious inhibitory activities against yeast α-glucosidase [9]. Chlorogenic acid, a phenolic compound found widespread in plants, is widely recognised as an antioxidant [29], which is a scavenger for reactive oxygen species [30]. Moreover, many studies have suggested that chlorogenic acid has hypoglycaemic effects [31–34]. It also has been identified as a novel insulin sensitiser that prompts insulin action similar to the therapeutic effect of metformin [35].The three chlorogenic acid derivatives were also reported to be active principles related to hypoglycemic effect [36]. The four bioactive components have vicinal hydroxyl groups on an aromatic residue [37], and the C = C linked to the phenyl ring may play a important role in stabilizing the radical by resonance which ensures their antioxidant capacity [38, 39]. It is generally known that the diabetogenic action of STZ can be illustrated as free radical caused toxicity particularly to pancreatic β-cells. And GD as an antioxidants, are considered to be the potential cure for diabetes [40]. Thus, with this background, we could conclude that chlorogenic acid and its derivatives, the major constituents of GD methanol extract, are possibly responsible for the anti-diabetic effect of oral GD lyophilized powder.

As is well known, diabetes is primarily caused by insulin resistance and islet dysfunction [41]. Insulin resistance is a hyperinsulinic condition in which insulin reduces blood glucose inadequately, causing higher sugar levels in plasma. The therapeutic strategies used to treat diabetes mainly focus on reducing and controlling blood glucose and recovering insulin level. The present results show evident declines in the FBG and GSP levels in diabetic mice after GD administration. GD also effectively reversed abnormal insulin levels in diabetic mice. The findings demonstrate that GD can effectively alleviate glycaemia and insulin resistance and enhance insulin sensitivity in type 2 diabetic mice. This was further supported by HOMA-IR and ISI.

OGTT is a kind of criterion to judge glucose tolerance. Insulin resistance is an early condition of metabolic disorders, which could lead to diabetes, hyperlipidemia and steatosis [42]. The above results show that GDP administration could improve glucose and insulin metabolism.

As is well known, STZ could cause oxidative stress injury in the pancreatic tissues. Importantly, GD markedly increased the activities of T-SOD and GSH-PX, and reduced the MDA production in the pancreatic tissue of diabetic mice, noting that GD treatment could scavenge oxygen free radicals directly and protect the pancreatic tissue against oxidative stress injury.

In the present investigation, the pancreas exhibited evident recovery of both the pathological changes in the pancreatic islets and the numbers of β-cells following treatment with GD. Simultaneously, this further supported by the result of immunohistochemical examination.

PDX-1 plays a vital role in pancreas development and retaining mature β-cell function [43]. In mature β-cells, PDX-1 is an important transcription factor for insulin gene expression [44]. PDX-1 transactivates insulin and other genes involved in glucose metabolism, such as GLUT2 and GK [45]. Our present study showed that the administration of GD significantly enhanced the expression of GLUT2, GK, and PDX-1 protein in diabetic mice, which indicated that GD treatment might cause increased the level of PDX-1, thereby transactivating the expression of GK and GLUT2. MafA, an efficacious activator of insulin gene transcription, may be a novel target for the treatment of diabetes [42]. Our results showed that GD effectively upregulated the level of pancreatic MafA.

Apoptosis is a significant biological process involved in the occurrence and development of various diseases including diabetes [46]. In general, the activation of caspase-3 could stimulate other caspases and ultimately induce cell apoptosis [47]. In order to further explore the relevant mechanism about that GD inhibited the apoptosis of β-cells, the potential role of GD on Bcl-2, Bax, and caspase-3 related to apoptosis were estimated in type 2 diabetic mice. Our present study indicated that the administration of GD significantly reduced the expression of Bax and increased the expression of Bcl-2 in the pancreas of diabetic mice. Also, GD treatment lower the level of caspase-3 in pancreatic islet cells. These findings stated that the potentially anti-apoptotic function of GD.

## Conclusion

All in all, our attractive results suggest that the treatment of GD rich in chlorogenic acid and its derivatives can successfully ameliorate hyperglycaemia and hyperinsulinemia and improve the function of the pancreas, which demonstrates that GD functions as a promising food or medicine for diabetes treatment by restoring pancreatic function effectively.

## Abbreviations

Bax: Bcl2-associated X
Bcl-2: B-cell lymphoma-2
caspase-3: cysteinyl aspartate specific proteases-3
CID: Collision-induced dissociation
FBG: Fasting blood glucose
GD: *Gynura divaricata*
GK: Glucokinase
GLUT2: Glucose transport protein 2
GSH-PX: Glutathione peroxidase
GSP: Glycosylated serum protein
HE: Hematoxylin-eosin
HFD: High-fat diet
HOMA-IR: Homeostasis model assessment of insulin resistance
HPLC: High-performance liquid chromatography
HPLC–DAD: High-performance liquid chromatography with diode array detection
ISI: Insulin sensitivity index
ITT: Insulin tolerance test
MafA: v-maf musculoaponeurotic fibrosarcoma oncogene family protein A
MDA: Malonaldehyde
OGTT: Oral glucose tolerance test
PDX-1: Pancreatic duodenal homeobox-1
SOD: Superoxide dismutase
STZ: Streptozotocin
T2DM: Type 2 diabetes mellitus
TCMH: Traditional Chinese medicinal herbs
